# Preliminary Evidence to Support a De-Escalated Cochlear Implant Programming Paradigm for New Adult Recipients: A Systematic Review

**DOI:** 10.3390/jcm12185774

**Published:** 2023-09-05

**Authors:** James R. Dornhoffer, Karl R. Khandalavala, Teresa A. Zwolan, Matthew L. Carlson

**Affiliations:** 1 Department of Otolaryngology-Head and Neck Surgery, Mayo Clinic, Rochester, MN 55905, USA; dornhoffer.james@mayo.edu (J.R.D.); khandalavala.karl@mayo.edu (K.R.K.); 2 Department of Otolaryngology-Head and Neck Surgery, Michigan Medicine, Ann Arbor, MI 48109, USA; tzwolan@cochlear.com; 3 Cochlear Americas, Denver, CO 80124, USA; 4 Department of Neurosurgery, Mayo Clinic, Rochester, MN 55905, USA

**Keywords:** cochlear implant (CI), programming, evidence-based, de-escalated

## Abstract

**Background:** No standard schedule for cochlear implant (CI) programming has been developed, and common practices may have CI recipients seen in excess of what is necessary. The objective of this study was to review evidence for a de-escalated, evidence-based schedule for adult CI programming. **Methods:** Systematic review was undertaken in March 2023 of PubMed, Scopus, and CINAHL databases using the Preferred Reporting Items for Systemic Reviews and Meta-analyses (PRISMA) guidelines. Studies were included if (1) they evaluated an evidence-based programming/follow-up schedule in new adult CI patients or (2) they evaluated programming or outcomes in a longitudinal fashion such that they could inform CI follow-up strategies. Level of evidence was evaluated using the LEGEND evidence assessment tool. **Results:** Our review identified 940 studies. After screening with a priori inclusion criteria, 18 studies were ultimately included in this review. Of these, 2 demonstrated feasibility of de-escalated approaches to new adult CI programming. The remainder presented longitudinal speech and programming parameter data that demonstrated relative stability of both categories by 3 to 6 months post-activation. **Conclusions:** Overall, there is a paucity of literature evaluating any form of evidence-based CI programming or follow-up. Most applicable data derive from longitudinal outcomes featured in studies of other CI features, with only a handful of studies directly evaluating CI programming strategies over time. However, stability in outcomes and programming detailed in the available data supports consideration of a de-escalated programming paradigm that could primarily limit programming to the very early post-activation period (before 3 to 6 months) to enhance patient care and reduce operational strains on cochlear implant programs.

## 1. Introduction

Cochlear implants (CIs) have become the standard of care for moderate to profound sensorineural hearing loss [1,2]. Once implanted, a recipient must undergo programming to tailor their CI to comfortable use and maximal benefit. This programming process involves manipulating upper level amplitudes of stimulation (also called comfort levels or C-levels) and may involve manipulation of threshold (T) levels while providing strategies for effective use of the CI [3]. A survey of major CI centers showed substantial variability in programming schedules and practices among programs both within the United States and internationally [4]. This survey demonstrated that many institutions favored frequent programming, sometimes exceeding 10 sessions in the first year, and continuing to see patients in perpetuity. However, the effectiveness or necessity of CI programming protocols that entail frequent patient visits for extended durations is unknown.

Regardless of efficacy, the frequency and timeline for CI programming has both financial and functional impacts. For patients, frequent programming sessions necessitate commutes to large, centralized CI centers. For healthcare centers, programming sessions are often poorly reimbursed, and the availability of programming audiologists is often outpaced by growing practices [5,6,7]. Additionally, frequent programming after the initial post-activation period may provide little benefit for patients, as many CI users approach their performance plateau early in the post-activation period [8]. Similarly, for many patients, changes to programming after 3 months are often minor [9]. As such, manipulating CI settings beyond 3 months, after a reasonable sound processor program has been established, may offer limited benefit and could be detrimental in some cases. Anecdotally, an experienced otologist who received a CI reported improved adaption to stimulation after cessation of frequent reprogramming [10]. The limited continued benefit from frequent programming sessions may be due to a limitation of the brain’s neuroplasticity to adapt to a ‘moving target’.

From the lack of evidence-based CI programming paradigms, the concept of “de-escalated” programs has evolved. In de-escalated programs, patients undergo fewer programming sessions at early activation timepoints, to maximize programming benefits and minimize resource utilization. Recently, Porps et al. [11] reported on an evidence-based programming paradigm that followed a small group of patients over a limited follow-up schedule of four programming visits after implantation. These patients showed appropriate improvements in speech as well as high levels of patient satisfaction. Unfortunately, such evidence to support similar initiatives remains scarce.

This study aimed to systematically review the literature for studies that evaluate evidence-based CI programming or studies that provide longitudinal data on speech outcomes or programming characteristics that could help inform an evidence-based CI programming timeline. This information could provide valuable data to develop streamlined and cost-effective CI patient care.

## 2. Material and Methods

### 2.1. Search Strategy

A literature search was performed following the Preferred Reporting Items for Systemic Reviews and Meta-analyses (PRISMA) guidelines [12]. Inclusion criteria were generated using the Participants, Intervention, Control, Outcomes, and Study designs (PICOS) strategy. PICOS inclusion criteria are detailed in Table 1 and broadly include studies evaluating an evidence-based programming/follow-up schedule or algorithm for new adult CI recipients or examining new adult CI programming parameters or speech outcomes in a longitudinal fashion with sufficient data to inform an evidence-based programming paradigm [13]. Specific exclusion criteria included lack of full-text availability, non-English language manuscript, pediatric population, lack of follow up to or beyond 6 months post-activation, and failure to report programming or speech recognition changes over time.

### 2.2. Study Identification

**Figure 1:**
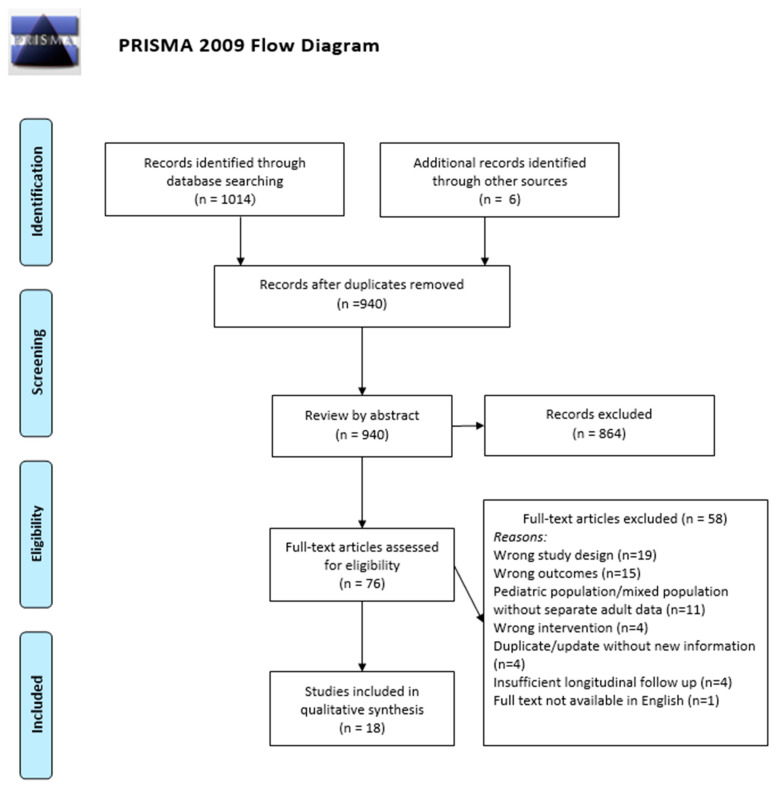
PRISMA flow diagram of systematic review.

A flow diagram of the study identification and review is detailed in Figure 1. Two reviewers (JD and KK) independently searched the PubMed, Scopus, and CINAHL databases in March 2023 for appropriate studies. The following search terms were used: cochlear implant OR cochlear implantation OR cochlear implants AND programming OR programs OR follow-up OR mapping OR C-level OR comfort level OR T-level OR threshold OR M-level AND de-escalation OR reduction OR strategy OR evidence-based OR scheduling OR fitting. The following filters were employed: English language and full text. Our search yielded 1014 articles with 6 additional articles added during review of included studies. After removing duplicate articles, 940 unique articles were identified.

### 2.3. Study Screening and Selection

Articles identified in our search were reviewed by title and abstract for our PICOS inclusion and exclusion criteria. There was no time range or limitations on publication date. After review by title and abstract, 76 articles underwent full-text review for inclusion, resulting in 18 articles included in this review [8,9,11,14,15,16,17,18,19,20,21,22,23,24,25,26]. Reasons for dismissal of full-text articles included the following: wrong study design (n = 19), wrong outcome measure (n = 15), pediatric population/mixed population without separate adult data (n = 11), wrong intervention (n = 4), duplicate article/update without new information (n = 4), insufficient longitudinal follow up (n = 4), full text not available in English (n = 1). Articles with overlapping patient populations were included if new information or analysis was made available. Namely, follow-up studies were included if they detailed new outcomes or programming patterns not detailed in a prior manuscript, or detailed follow-up beyond the scope of the prior publication. Disagreements among reviewers were mediated by the senior author (MC).

### 2.4. Data Extraction

Manuscripts were reviewed and data extracted by two independent reviewers (JD and KK). Discrepancies were resolved by a senior author (MC). Data collected from each manuscript included author, year of publication, study design, patient population and details, device manufacturer, bilateral vs. unilateral implantation, speech outcome evaluated and notable findings, and programming measure evaluated and notable findings. Level of evidence was evaluated using the LEGEND (Let Evidence Guide Every New Decision) evidence assessment tool for mixed modality studies [27]. LEGEND is an appraisal tool developed at the University of Cincinnati for analysis of the body of evidence from a wide variety study of formats (e.g., prospective retrospective, qualitative, and quantitative designs). Per the use of this appraisal tool, the body of literature is ranked from a very low to high grade of evidence based on the number and quality of individual studies.

## 3. Results

### 3.1. Study Characteristics and Populations

Study characteristics are summarized in Table 2. A total of 18 publications were identified that met a priori inclusion and exclusion criteria. Publication dates ranged from 2001 to 2023. The majority of studies (n = 13) were repeated measure studies, without any control, namely observation of speech outcomes or programming parameters over time. Only 2 of the 18 studies, Porps et al. [11] and Zwolan et al. [25] primarily evaluated a specific programming or follow-up strategy. The remaining studies either directly or indirectly evaluated speech outcomes or programming parameters over time for new adult CI recipients. Of these, 5 studies focused on outcomes or comparisons beyond the scope of outcomes or programming parameters over time but reported sufficient longitudinal data that could be used in this study [14,15,21,26,28].

Characteristics of the study populations are also detailed in Table 2. Patient populations ranged widely from 10 to 804 patients. Studies that provided such data indicated that all three United States Food and Drug Administration (FDA)-approved CI manufacturers (Advanced Bionics Corp. (Valencia, CA, USA), Cochlear Ltd. (Sydney, Australia), and MED-EL GmbH (Innsbruck, Austria)) were represented in the literature and most implantations were unilateral. Using the LEGEND evidence assessment tool for mixed modality studies, the overall level of evidence for this body of research was low [27]. Levels of evidence for individual studies were poor given the predilection for uncontrolled cohort studies, and many studies were downgraded given the indirect nature of the outcomes that were collected.

Given that most studies had data on either speech outcomes or programming parameters over time, we considered each data set separately. We then considered the two studies that evaluated a specific programming or follow-up strategy last.

### 3.2. Speech Outcomes over Time

Of included studies, 13 reported on at least one measure of speech recognition in new adult CI recipients, Table 3 [8,9,11,14,15,16,17,18,21,24,25,26,28,29]. These included CI soundfield thresholds, mono/multisyllabic word recognition, Consonant-Nucleus-Consonant (CNC) word score [30], Freiburger monosyllabic test score [31], AzBio Sentences score [32], Hochmair-Schulz-Moser (HSM) sentence score [33], and Hearing in Noise Test (HINT) score in various levels of signal-to-noise ratios [34]. Aimoni et al. [14] reported on speech recognition categories. Caswell-Midwinter et al. [16] and Frijns et al. [18] each reported on time to speech recognition plateau, at a median of 2.9 months [IQR: 0.9–9 months] and 3 months, respectively. Hilly et al. [21] reported on HINT stability (<20% change), with no change from 1-year post-activation to a patient’s most recent follow-up, at an average of 6.8 years. For speech tracking over time, Frijns et al. [18] demonstrated a peak at 3 months with a slight drop-off at 6 months, while Lenarz et al. [28] demonstrated a plateau at 3 months without significant changes to speech tracking out to 5 years. Universally, we saw that the greatest magnitude of improvement in all outcomes occurred between preimplantation/activation and 1 month, when reported, or between preimplantation/activation and 3 months.

Most studies supported these findings of significant improvements in time intervals up to 3 months and without continued significant change beyond the initial post-implant time frame. However, several notable exceptions apply. For CNC word scores, Kelsall et al. [29] demonstrated continued significant, albeit reduced, improvement at time intervals up to 12 months. Porps et al. [11] reported continued significant improvement up to 6 months post-activation. Additionally, Lenarz el al. [28] reported gradual improvement in HSM sentence scores up to 2 years post-activation; however, statistical analysis was not performed. Finally, Ruffin et al. [24] demonstrated that time to maximal score on speech recognition using a combination of NU-6 and CNC word lists ranged from 9 to 12 months for their cohort of 31 CI recipients. Grisel et al. [8] reported on the largest group of patients, 804 CI recipients from the HERMES database, and demonstrated that the largest improvements are seen between preimplantation and 1 month and then between 1 and 3 months post-implantation, with continued significant, but smaller, improvements beyond 3 months. This large database study also stratified patients into high and low performers based on CNC scores at or beyond 3 months and concluded that patients reaching CNC scores > 50% should be considered for de-escalated follow-up given that scores in this group are largely stable beyond 3 months.

Considering the durability of outcomes with time, Hilly et al. [21] reported outcomes out to 6.8 years post-activation with no significant reduction in HINT scores during that time. Lenarz et al. [28] similarly reported stability in speech recognition out to 5 years, and Ruffen et al. [24] reported stable speech recognition out to 10 years post-activation.

### 3.3. Programming Parameters

Seven of the included studies reported on at least one measure of speech recognition in new adult CI recipients, Table 4 [9,16,17,19,20,22,23]. All studies detailed T- and C-levels in some fashion. Caswell-Midwinter et al. [16] did not analyze parameters over time but did analyze the association of initial T- and C-level with time to performance plateau, although no significant associations were found. Domville-Lewis et al. [17] reported on time to parameter stability, defined either by lack of statistical or clinical change (as determined by a senior audiologist at their institution) for sequential bilateral CIs. This study demonstrated that mean time to programming stability was 77.6 days and 87.3 days, as defined statistically and clinically, respectively, for a first implant, and 57.8 days and 50.6 days, respectively, for a second implant.

The remaining studies, as with the studies on speech outcomes, generally showed greatest changes in the first 3 to 6 months following implantation/activation, with decreased modifications beyond the initial post-implantation period. Studies detailing notable changes beyond 6 months include Hughes et al. [22], who demonstrated continued increase in C-levels over the first 12 months but no change in T-levels from initial stimulation, and Wathour et al. [9], who examined programming practices between four CI centers and reported that two centers did not reach steady parameter settings until 1 year after activation; however no statistical analysis was performed on these parameters over time.

Considering the long-term stability of parameters, Gajadeera et al. [19] reported T- and C-levels up to 10 years post-activation and found that 75% of patients showed less than 6% change in parameters each year as a function of dynamic range (the difference between T- and C-levels). Patients who did have changes were equally likely to have a decrease or increase in level.

### 3.4. Evidence-Based Programming Trials

As detailed previously, only two studies directly evaluated an evidence-based or de-escalated programming paradigm [11,25]. Zwolan et al. [25] studied the effects of using a computer-guided programming system, titled “Fitting to Outcome eXpert” for FOX (Otoconsult, Antwerpen, Belgium). By using this program, they were able to reduce the number of programming visits by 43% compared to a survey of CI centers on their general practices, while maintaining similar CNC and AzBio scores at 6 months post-activation compared to a cohort of patients with identical implant models. Porps et al. [11] examined the effects of a de-escalated programming strategy, reducing programming sessions to four visits (activation, 1, 3, and 6 months post activation). With the de-escalated programming timeline, 82% of participants were able to keep to the reduced schedule, with only three patients requiring additional visits. Of these three patients, only one required additional visits related to programming changes. As a group, patients reported excellent satisfaction with the programming service and high proportions of satisfaction with their CI hearing outcomes at 6 months, with the exception that satisfaction was not increased in the category of “background noise”. CNC scores showed significant improvements from preimplantation to 3 months and subsequently from 3 months to 6 months. No comparisons were made between normally programmed peers. Porps et al. [11] also employed Remote Check, a self-diagnostics program, and a Recipient Solutions Manager, a Cochlear Americas team member who helps facilitate access to questions and materials (Cochlear Americas, Lone Tree, CO, USA). The impact of these additional tools on hearing outcomes or overall visit reduction was not established; however, survey results demonstrated that 71% of patients reported they were likely to use Remote Check, and 41% interacted with a Recipient Solutions Manager during the CI programming period.

## 4. Discussion

Post-surgical care for CI recipients is an evolving process, during which a recipient must undergo programming to tailor their CI to facilitate optimal use. Unfortunately, there exists no standardized algorithm for programming in adults, with many centers favoring frequent programming during the first 1 to 2 years, often with annual programming in perpetuity [4]. Without an evidence-based CI programming strategy, the effectiveness or necessity of the current programming structure remains unknown. Given that the median catchment area of many CI centers is 52 miles, which extends farther for more rural recipients, these frequent programming sessions may impose a major burden with respect to both patient time and finances [6]. Furthermore, frequent programming introduces financial and operational strains for CI centers given current reimbursement and often limited bandwidth of programming audiologists at larger established CI centers.

In this study, we have reviewed the extant literature on evidence-based paradigms of implant programming as well as studies that detailed speech outcomes and programming parameters over time in new adult CI recipients. In doing so, we have demonstrated that both speech outcomes and CI programming parameters are largely stable by 3 to 6 months post-activation. While some studies demonstrate continued improvement in speech scores up to a year or more after activation, these changes are generally small and could be explained by ongoing learning processes or brain plasticity rather than changes to programming. The decreasing margin of benefit at longer timepoints is also supported by the fact that most programming changes (e.g., change in T- and C-levels) stabilize around 3 months [9,16,17,19,20,22,23]. However, much of these data remain limited given the overall poor level of evidence from this body of literature. Only two studies more directly examined streamlined programming paradigms, and while they demonstrated favorable outcomes amongst patients following these de-escalated strategies, follow-up was limited as data were reported out to only 6 months [11,25].

In reviewing the current body of literature, CI centers can consider a streamlined and more flexible paradigm of CI programming and follow-up that focuses on rapid acquisition of a stable CI programming map and then transitioning to as-needed follow-up. Given the patterns of speech acquisition and programming changes detailed in this study, Table 3 and Table 4, a de-escalated strategy would aim to stop programming around 3 to 6 months for most patients. Domville-Lewis et al. [17] reported a median time to a stable map of around 70 to 90 days for new implant recipients. After this period, patients would be expected to use and practice with their implant, which would likely yield continued improvement as the brain adapted or learned to hear with the CI, perhaps explaining some of the slower but significant speech gains seen in some of the patient populations in this study [35,36]. Additional interactions beyond the 3-to-6-month time frame could be via telemedicine or electronic messaging in order to encourage CI use and facilitate contact with the center on an as needed basis. Given the stability in both programming parameters and speech outcomes demonstrated in the current review, long-term CI follow-up will likely be enhanced if patients are primarily seen when new equipment or software upgrades are available or on an as-needed basis [37].

However, we must consider that while many of these considerations are suitable at the population level, they may not be appropriate for each individual patient. For example, patients with unique device needs, such as those affected by the Advanced Bionics V1 recall, should be followed more closely than the general implant population, as stability of programming and outcomes is less assured [38,39]. Patients with initial poor performance may also not be good candidates for de-escalated programming. As demonstrated by Grisel et al. [8], many- to most patients derive significant early benefit that is largely stable beyond 3 to 6 months, but a portion of poor performers may have continued change in performance up to 12 months or more after activation. Patients with initial poor performance may therefore benefit from more frequent programming over a longer period of time. As such, CI providers way wish to only pursue de-escalated programming in suitably well performing CI recipients. Grisel et al. [8] suggested a CNC word score of 50% or more at 3 months as a cut off for patients that are suitable for de-escalated programming, while Porps et al. [11] utilized a cut off of >20% improvement in CNC word score at 3 months and >30% by 6 months when pre- and post-implant scores were compared, for inclusion in their evidence-based programming trial. Continued research may be needed to identify an ideal metric to identify patients who are appropriate for de-escalated programming.

Providers may also be concerned that de-escalated programming could result in a failure to identify patients that have a decline in performance after initially doing well. Anecdotally we have found that more patients self-report issues with their device than are found to have problems during a routine visit, and, as such, do not feel this to be a barrier to reduction in programming visits. However, this concern can be also assuaged through the use of technologies such as the digit triplet test and resources for remote care [40]. In the era of COVID, telemedicine saw a dramatic rise in both use and institutional/insurance support. While sometimes challenging for patients with hearing impairment, telemedicine and alternative options for remote care have been demonstrated as feasible in the CI population [41]. Additionally, tools have been created to further enhance access to remote care or self-diagnostics. For example, one manufacturer has released Remote Check, which allows CI users to perform diagnostic and speech testing that can be viewed or sent to a provider without in-person contact and also supports the use of a Recipient Solutions Manager program, which provides access to real time advice, training, and trouble shooting. These programs, and other such initiatives, may help to support a streamlined-programming paradigm, or at least help develop a more patient-centered and directed care model that more efficiently uses both patient and CI center resources. However, further study is still needed to determine patient engagements with such resources and their real-world effectiveness.

Our present study has several limitations. The current level of evidence for this body of literature is low, with very limited direct evaluation of follow-up strategies. As such, further studies with elongated follow-up time points are required, in excess of 1 to 2 years. A controlled trial comparing a streamlined paradigm of programming to routine practice would be essential. Any such trial could also employ telehealth and remote technology opportunities currently available in order to model current available care. While the current body of literature offers limited data, we believe that it allows for consideration of changes to traditional CI care.

Recently, a modified Delphi consensus process was used to revise the Minimum Speech Test Battery (MSTB) to create the MTSB-3 (Dunn and Zwolan, submitted for publication). A consensus process was needed to develop recommendations regarding CI care since, as mentioned above, data to support evidence-based recommendations regarding CI Care is lacking. Similar to our conclusions following this systematic review, the MSTB-3 recommends a de-escalated schedule for post-operative assessment of performance. Previous versions of the MSTB included recommendations for CI evaluations to occur 1, 3, 6, and 12 months post-implant, plus annual monitoring of performance thereafter. The MSTB-3 recommends baseline testing of outcomes at 3 months, and only recommends re-evaluation at 6 months if the clinician feels the patient is not making adequate progress. It additionally recommends testing at 12 months, with subsequent evaluations being performed on an as-needed basis rather than annually.

## 5. Conclusions

Programming of new adult CI users can be a complex and time-consuming process, with no standardized schedule. This systematic review of the literature identified 18 studies with information on programming schedules and on CI program and speech perception outcome stability in new adult implant recipients. These studies demonstrate that CI programming parameters and speech outcomes generally begin to stabilize within the first month post-activation. Additionally, two studies demonstrate the feasibility of a de-escalated programming schedule for new adult recipients. While this body of literature is limited by a low level of evidence, it provides preliminary support for consideration of new, streamlined CI care, with reduced frequency and duration of programming for patients meeting a certain audiological standard at certain time points. Further study is necessary to better define this threshold and overall care algorithm.

## Figures and Tables

**Table 1:** PICOS inclusion criteria for systematic review.

Participant	New cochlear implant recipient, implanted as an adult (≥18 years)
Intervention	Any de-escalated or evidence-based scheduling of follow-up and/or programming; or any examination of follow-up, programming, or outcomes over the first 6 months or more post-activation to inform the above
Control	Natural or observational studies did not require a control; studies examining specific modifications in follow-up or scheduling were compared to normal control or historical implant data
Outcome	Subjective implant function, objective speech testing, quality of life, cost/time savings, and/or programming parameters
Study	Randomized controlled trials, non-randomized controlled trials, cohort studies with control groups, and repeated measure studies (including single sample case studies)

**Table 2:** Study characteristics and populations.

Study Details		Implanted Patient Population			Implant Details		LEGEND Grading
Author Year	Study Design	N	Age in Years (Avg (Range))	Gender (% Female)	UL/BL	Company (% Cohort)	
Aimoni C 2016 [14]	Case-control comparing elderly and nonelderly outcomes; indirect repeated measures data is available on outcomes over time	57	Elderly group: 77 (65–86) Nonelderly group 50 (40–49)	53%	100% UL	Cochlear (NA) Advanced Bionics (NA) MED-EL (NA)	4b
Bruschke S 2021 [15]	Controlled cohort comparing early and normal date of activation; indirect repeated measures data is available on outcomes over time	127	Early activation: 63 (22–88) Normal activation: 61 (27–81)	50%	91% UL 9% BL	Cochlear (65%) Advanced Bionics (6%) MED-EL (39%)	4b
Caswell-Midwinter B 2022 [16]	Repeated measure analysis of a large database	88 *	63 (24–93)	NA	89% UL 11% BL	Advanced Bionics (100%)	4a
Domville-Lewis C 2015 [17]	Repeated measure analysis of stability of CI maps	33	First implant, 48 (1–79) Second implant 51 (2–81)	57%	100% BL	Cochlear (100%)	4a
Frijns JH 2002 [18]	Repeated measure analysis of CI outcomes	10	44 (14–62)	70%	100% UL	Advanced Bionics (100%)	4b
Gajadeera E 2017 [19]	Repeated measure analysis of T and C levels	128	59 (19–85)	NA	NA	Cochlear (100%)	4a
Gajadeera E 2017 [20]	Repeated measure analysis of T and C levels	680	59 (19–93)	NA	NA	Cochlear (100%)	4a
Grisel J 2022 [8]	Repeated measure analysis of HERMES CI database	804	Database: NA (18–109)	NA	100% UL	Cochlear (80%) Advanced Bionics (11%) MED-EL (9%)	4a
Hilly O 2016 [21]	Controlled cohort comparing elderly and nonelderly CI recipients; indirect repeated measures data is available on outcomes over time	87	32–≤ 60 years 33–61–70 years 22–>70 years	62%	90% UL 10% BL	Cochlear (36%) Advanced Bionics (59%) MED-EL (5%) †	4b
Hughes ML 2001 [22]	Repeated measures analysis of T and C levels	35	53 (29–77)	NA	83% UL 17% BL	Cochlear (100%)	4a
Kelsall D 2021 [29]	Repeated measures analysis of CI outcomes	100	67 (23–93)	37%	100% UL	Cochlear (100%)	4a
Lenarz M 2012 [28]	Controlled cohort comparing male and female CI outcomes; indirect repeated measures data is available on outcomes over time	638	5 (no range provided)	56%	100% UL	NA	4b
Mosca F 2014 [23]	Repeated measures analysis of CI fitting parameters	26	no average (18–58)	39%	100% UL	Cochlear (100%)	4a
Porps SL 2023 [11]	Repeated measures analysis of CI outcomes and satisfaction with a streamlined programming strategy	17	62 (24–80)	NA	94% UL 6% BL	Cochlear (100%)	4a
Ruffin CV 2007 [24]	Repeated measures analysis of CI outcomes	31	51 (25–74)	58%	100% UL	Cochlear (100%)	4b
Wathour J 2021 [9]	Cross-sectional comparison of fitting practices between 4 CI centers	97	Center 1: 55 Center 2: 54 Center 3: 51 Center 4: 58	NA	NA	Cochlear (100%)	4a
Zwolan TA 2021 [25]	Repeated measures analysis of CI outcomes after fitting with computer-assistance	31	63 (23–90)	48%	100% UL	Cochlear (100%)	4a
Zwolan TA 2001 [26]	Repeated measures analysis of CI outcomes and comparison between pre-curved and straight electrodes with electrode positioning systems; indirect repeated measures data is available on outcomes over time	112	First cohort: 54 ± 16 Second cohort: 61 ± 16	NA	100% UL	Advanced Bionics (100%)	4b

RCT = randomized controlled trial; non-RCT = non-randomized controlled trial; CI = cochlear implant; T-level = threshold level; C-level = comfort level; NA = not available; UL = unilateral; BL = bilateral. * Sample size included for review, 384 patients in initial study. † Percentage cannot account for bilateral implants.

**Table 3:** Longitudinal speech outcomes.

Author Year	Speech Outcome Measure(s) over Time	Results
Aimoni C 2016 [14]	CI pure tone thresholds	Significant improvements from preop to 1 month, no significant improvement from 1 month to 12 months.
	Speech perception performance category	Significant improvements in perception category from preop to 1 month. Significant improvement in category allocation from 1 month to 12 months.
Bruschke S 2021 [15]	Multisyllabic word score	Significant improvement from preop/activation to 3 months; no significant change from 3 months to 6 or 12 months.
	Monosyllabic word score	Significant improvement from preop/activation to 3 months; no significant change from 3 months to 6 or 12 months.
Caswell-Midwinter B 2022 [16]	Time to word recognition score plateau (CNC word)	Median time plateau score of 2.9 months [IQR = 0.9–9.0 months], Median plateau score of 61.2% [IQR = 46.8–71.3%],
Frijns JH 2002 [18]	Word recognition testing with CVC word lists-phonemes reported	Score plateau at 3 months: 80% phoneme, 62% word. Avg last available score: 84% phoneme, 66% word. †
	Speech tracking (words per minute)	Peak at 3 months (66 words per minute), slight drop of at 6 months and no data beyond 6 months.
Grisel J 2022 [8]	CNC word	Individually, significant improvements were seen at each interval up to 12 months, with the largest changes between preimplantation and 1 month, and 1 month and 3 months. Highest CNC word score: 76.7% achieved between 3 and 12 months after activation.
Hilly O 2016 [21]	HINT score stability *	No patients with deterioration > 20% after 1 year. 13.6% of patients older than 70 showed continued improvement.
Kelsall D 2021 [29]	CNC word	Significant improvement from preimplantation to 3, 6, and 12 months. Significant improvement between intervals; greatest interval of improvement from preimplant to 3 months (41.8%) with lesser improvement from 3 to 6 months (4.6%) and 6 to 12 months (3.4%).
	AzBio sentences in +10 SNR	Significant improvement from preimplantation to 3, 6, and 12 months. Significant improvement between 3 months and 6 and 12 months; greatest interval of improvement from preimplant to 3 months (19.1%) with lesser improvement from 3 to 6 months (8.8%) and 6 to 12 months (3.2%).
	AzBio sentences in +5 SNR	Significant improvement from preimplantation to 6, and 12 months (3 months data not collected). No significant improvement between 6 and 12 months; greatest interval of improvement from preimplant to 6 months (10.9%) with lesser improvement from 6 to 12 months (3.2%).
Lenarz M 2012 [28]	Freiburger monosyllabic Test	No statistical analysis on repeated measures over time; qualitative analysis shows largest increased from implantation to 3 months with small gradual increase to 1 year with stable scores up to 5 years.
	Speech tracking test	No statistical analysis on repeated measures over time; qualitative analysis shows largest increased from implantation to 3 months with stable scores up to 5 years.
	HSM sentence test in quiet	No statistical analysis on repeated measures over time; qualitative analysis shows largest increased from implantation to 3 months with small gradual increase to 2 years with stable scores up to 5 years.
	HSM sentence test in −10 SNR	No statistical analysis on repeated measures over time; qualitative analysis shows the largest increased from implantation to 3 months with small gradual increase to 2 years with stable scores up to 5 years.
Porps SL 2023 [11]	CNC word	Significant improvement from preimplantation to both 3 and 6 months post activation with significant improvement between 3 and 6 months; greatest interval of improvement from preimplant to 3 months (53.3%) with lesser improvement from 3 to 6 months (9%).
	AzBio sentence test in +10 SNR	Significant improvement from preimplantation to 3 and 6 months post activation with no significant difference between 3 and 6 months; greatest interval of improvement from preimplant to 3 months (31.2%) with lesser improvement from 3 to 6 months (5.3%).
Ruffin CV 2007 [24]	Speech recognition with a combination of NU-6 and CNC word lists	Most significant growth in performance noted in first 9 months; time to maximum score ranged from 9 months to 120 months for the whole cohort.
		Analysis of performance after 24 months shows no significant change beyond this timepoint.
Wathour J 2021 [9]	CI pure tone thresholds	No longitudinal analysis; thresholds at 1 year post activation without significant difference between centers-despite programming differences.
	Speech recognition testing	No longitudinal analysis: scores provided for 2 centers but disparate tests prevent comparison.
Zwolan TA 2021 [25]	CNC word	Significant improvements from preimplantation to 3 and 6 months; no significant difference between 3 and 6 months
	AzBio	No preoperative measures; no significant difference between 3 and 6 months.
Zwolan TA 2001 [26]	CNC words CID sentences HINT in Quiet HINT +10 SNR	Significant improvement from preimplantation to 1 month; continued improvement from 1 to 3 months and 3 to 6 months but no significant difference. ‡

CID, central institute for the deaf; CNC, consonant-nucleus-consonant; HINT, hearing in noise test; HSM, Hochmair–Schultz–Moser; NA, not applicable; NU-6, Northwestern University-6; SNR, signal-to-noise ratio. * Defined as <20% change from 1 year post-implantation to most recent score (avg 6.8 years). † Data to 11 months available for 5 patients showing no notable change. ‡ Results applicable to all listed measures.

**Table 4:** Longitudinal programming parameters.

Author (Year)	Programming Parameter(s) over Time	Results
Caswell-Midwinter B 2022 [16]	Association of initial T- and C-level with time to performance plateau	Programming parameters were not significantly associated with time to plateau
Domville-Lewis C 2015 [17]	Time to CI map stability *	Mean days to stability: 77.6 ± 47.4 for first implant, 57.8 ± 28.2 for second implant, and 67.7 ± 39.9 for all implants
	Time to CI map stability †	Mean days to stability: 87.3 ± 53.9 for the first implant, 50.6 ± 24.6 for the second implant, and 69.0 ± 45.5 for all implants
Gajadeera E 2017 [19]	Mean T-level of all cochlear segments	18% of patients showed a significant trend in change over time from 6 months to 8–10 years; however, these trends were equally likely in either direction.
	Mean C-level of all cochlear segments	24% of patients showed a significant trend in change over time from 6 months to 8–10 years; however, these trends were equally likely in either direction.
		At least 75% of patients showed less than 6% change in C and T levels over from 6 months to 8–10 years as a function of dynamic range
Gajadeera E 2017 [20]	Mean T-level of all cochlear segments	Current level at 2 months significantly lower than compared to all time points up to 24 months; consecutive time points did not differ significantly after the 3-month time period
	Mean C-level of all cochlear segments	Current level at 2 and 3 months significantly lower than compared to all time points up to 24 months and current level at 24 months was significant higher compared to 6 and 12 months; consecutive time points did not differ significantly after the 6-month time period
Hughes ML 2001 [22]	MAP C-level	Significant improvement in the first 12 months; average increase in 11.8 programming units (30% of average dynamic range) over the first year. ‡
	MAP T-level	No significant change from initial stimulation to 24 months. §
Mosca F 2014 [23]	Average T-level	Significant change from preimplantation to 1 month and 1 month to 3 months; no significant change from 3 month to 6 or 12 months.
	Average C-level	Significant change from preimplantation to 1 month and 1 month to 3 months; no significant change from 3 month to 6 or 12 months.
Wathour J 2021 [9]	Average T-level	Significant different between centers at each time point; levels are stable after 3 months with low difference within centers after this point; no statistical analysis on levels over time but authors describe patients reaching steady state at 3 months for 1 center, 6 months for 1 center, and by 1 year for 2 centers.
	Average C-level	Significant different between centers at each time point; levels are stable after 3 months with low difference within centers after this point; no statistical analysis on levels over time but authors describe patients reaching steady state at 3 months for 1 center, 6 months for 1 center, and by 1 year for 2 centers.
	Maintenance of default manufacturer parameters at 1 year	Parameters that rarely changed from default values were programming strategy, stimulation mode, rate, pulse width, and loudness growth.
		Parameters that were more often changed from default values were T-SPL, C-SPL, and maxima.

C-level, comfort level; EAP, electrically evoked action potential; NA, not applicable; T-level, threshold level; SPL, sound pressure level. * Defined statistically as no greater than 10% change in all of C-level, T-level, and Dynamic Range. † Defined audiologically by visualization of scores by a senior audiologist. ‡ Impedance over time showed no changed after 1–2 months. § EAP thresholds stable since initial stimulation; EAP maximum slope increased from initial stimulation to 1–2 months and are stable afterwards.

## Data Availability

Not applicable given the literature review format of this study.
